# Prevalence of Hepatitis B Viral Infection in Pregnant Women at the Suhum Municipality, Ghana

**DOI:** 10.1155/2024/9438762

**Published:** 2024-04-01

**Authors:** Joseph Boachie, Doreen Pidah, Henrietta Eshun, Emmanuel Jingbeja, Praise Fosu Adjei, Patrick Adu

**Affiliations:** ^1^Department of Medical Laboratory Sciences, School of Allied Health Sciences, University of Health and Allied Health Sciences, Ho, Volta Region, Ghana; ^2^Department of Medical Laboratory Sciences, School of Allied Health Sciences, College of Health and Allied Sciences, University of Cape Coast, Cape Coast, Ghana; ^3^Department of Epidemiology and Disease Control, School of Public Health, University of Ghana, Legon, Accra, Ghana

## Abstract

**Background:**

Global prevalence of chronic hepatitis B virus (HBV) infection was estimated between 257 and 291 million since 2020, posing a great public health challenge. In Africa, an estimated 60 million cases of HBV were reported in the same year. Pregnant women might be susceptible to HBV infection dependent on their level of awareness and knowledge about the causes, transmission, and prevention of HBV. The aim of the study was to assess the awareness and prevalence of HBV infection and prolonged bleeding risk among pregnant women at the Suhum Municipality of Ghana.

**Methods:**

The study was a cross-sectional design involving pregnant women who were sampled following their visit to the antenatal unit at the Suhum Government Hospital. Sociodemographics including history of HBV screening and vaccinations were obtained from consented individuals using pretested questionnaires. Also, venous blood samples were obtained for platelet count, whereas bleeding time assay was performed to assess functional platelet disorders.

**Results:**

Hepatitis B prevalence was 4.4%, with 14.1% prevalence of mild thrombocytopenia and 1.5% prevalence of prolonged bleeding time. Pregnant women who had tertiary education and previous screening were about 8 times (AOR = 7.78, 95% CI: 1.50-40.50) and 14 times (AOR = 13.66, 95% CI: 1.72-108.75) more likely to have knowledge of hepatitis B than those without tertiary education and previous screening, respectively.

**Conclusion:**

The prevalence of HBV was 4.4%. Education status and previous screening were associated with demonstration of knowledge about HBV; therefore, intensification of education and screening are recommended.

## 1. Introduction

Infection with hepatitis B virus (HBV), an enveloped DNA virus, is a life-threatening disease of the liver that causes a major public health burden globally. In 2020, it was estimated that chronic HBV infection affected between 257 and 291 million of the global population, with its associated life-threatening complications including cirrhosis and carcinoma of the liver, as well as significant cases of morbidity and mortality [1]. Pregnant women are susceptible to HBV infection, and a Chinese national screening of about 90.87 million pregnant women within a 5-year interval reported 5.60 million (6.17%) positive cases for hepatitis B surface antigen (HBsAg) [2]. In Africa, an estimated 60 million cases of HBV were reported in 2020 [3]. Some local communities have reported diverse prevalence among pregnant women in West African countries including Ghana (6%) [4], Nigeria (8%) [5], and Burkina Faso (11.94%) [6]. In an African community, evidence of a decline in awareness and knowledge about HBV has been reported. Predominant factors such as cultural rules and religion were barriers that hindered HBV awareness and prevention, as well as accessibility to healthcare [3]. Vaccination against HBV is a paramount policy in public health to alleviate or combat the consequences resulting from the infection. The World Health Organization has targeted an estimated 90% global vaccination leading to the elimination of HBV by the year 2030 [7]. An estimation of HBsAg global prevalence was expected to be 5.2% (4.6-5.8) by the year 2019, an indication of about 402 million cases; however, interventions via vaccination accounted for the prevention of 85 million cases [8].

Thrombocytopenia is relatively acknowledged as a consequence of hepatic viral infection and might be considered the key manifestation of viral hepatitis [9]. In HBV infection, the pathogenesis of thrombocytopenia is not clearly understood; however, advanced cases are attributed to hypersplenism resulting from portal hypertension. Other less common mechanisms of thrombocytopenia in chronic viral hepatitis might be associated with impaired platelet production as a result of suppressed liver synthesis of thrombopoietin, aggravated platelet destruction, and damage to megakaryocytes and platelets by viruses [10]. Prolonged bleeding risk resulting from quantitative and functional defects of platelets poses a high risk to pregnancy outcomes, requiring prompt monitoring and treatment.

Despite the availability of vaccines, HBV remains a serious global health problem. In Ghana, the prevalence and risk factors of HBV infection as well as associated risks of prolonged bleeding/prothrombotic outcomes among pregnant women in several parts of the country remain unknown. The aim of this study was to assess the awareness and prevalence of hepatitis B viral infection and prolonged bleeding risk in pregnant women visiting the Suhum Government Hospital.

## 2. Materials and Methods

### 2.1. Study Design and Setting

The study was a cross-sectional hospital-based study in pregnant women. The study was designed to examine the prevalence and risk factors associated with HBV infection among pregnant women attending antenatal clinic at Suhum Government Hospital. The hospital is a referral center located in Suhum Municipal of the Eastern Region of Ghana. It provides antenatal and other specialized obstetric services for the inhabitants of the district and beyond and is predominantly a farming community. Suhum Municipality is located approximately 60 km from Accra, the national capital of Ghana. It is situated in the South Eastern part of the Eastern Region between latitude 0° 56 N and latitude 6° 08 N and longitude 0° 33 W and longitude 0° 16 W and covers a land area of about 359 square kilometers.

### 2.2. Sample Size and Sampling Method

The study was done among one hundred and thirty-five (135) pregnant women ≥ 18 years of age who were recruited using the convenient sampling approach. The sample size for this cross-sectional study was determined using the following formulae:

(1) $$\begin{array}{c} N=\frac{Z^{2}P\left(1-P\right)}{d^{2}},\\ N=\frac{{\left(1.96\right)}^{2}\times 0.095\left(1-0.095\right)}{{\left(0.05\right)}^{2}}. \end{array}$$

where *N* denotes the sample size, *Z* is the statistic (1.96) that corresponds to the degree of confidence, *P* is expected prevalence based on previous prevalence (9.5%), and *d* is the effect size or precision (0.05). Sample size was estimated to be 132.11. Therefore, a total number of 135 pregnant women were recruited.

The duration of the study was from August 2021 to January 2022. The study included questionnaire administration to the pregnant women for obtaining data relating to sociodemographics. Questionnaire administered was interpreted in the language understood by the participant to ascertain their knowledge about the disease.

### 2.3. Inclusion Criteria

The study exclusively involved pregnant women, aged ≥18 years who visited the antenatal clinic.

### 2.4. Exclusion Criteria

The study excluded women whose pregnancy status was not known and pregnant women who were diagnosed with other chronic liver diseases besides hepatitis B. Likewise, pregnant women with clinical records showing notable disorders such as immune/hereditary thrombocytopenia, eclampsia, preeclampsia with severe features, infections, and autoimmune disorders were excluded from the study.

### 2.5. Ethical Consideration

Ethical clearance was obtained from the Institutional Review Board (IRB) of the University of Cape Coast and the Research Ethics Committee (REC) of the University of Health and Allied Sciences with an approval ID: UCCIRB/CHAS/2022/202. Approval was also obtained from the administration and the laboratory department of Suhum Government Hospital. In addition, written consent was obtained from all participants who agreed to participate in the study.

### 2.6. Assessment of Participants' Knowledge about HBV

The study primarily assessed, via the administered questionnaires, the knowledge of pregnant women based on their general awareness of the infection (whether or not they had ever heard about HBV), the causes, and transmission as well as prevention strategies. In the event that participants answered “yes” indicating that they had knowledge about HBV, the causes, and mode of transmission, they were asked further to explain and/or give examples to validate their answers.

### 2.7. Laboratory Assays

Bleeding time assay was performed using the Ivy method explained by Mielke et al. [11]. Briefly, a blood pressure cuff was tied around the arm of the participants and adjusted to 40 mmHg. At a clear, 70% ethanol-disinfected region on the lower arm, two small horizontal 1 mm deep incisions were made with a lancet. The time taken for bleeding to completely stop after blotting the blood at the site of incisions was recorded using a stopwatch. Bleeding time between 2 and 9 minutes was utilized as normal range in the study [12].

Also, 4 mL of venous blood was collected from each participant through venipuncture, dispensed into an EDTA anticoagulated tube for platelet count estimation. This was determined using Sysmex XN-350-automated analyzer (USA). Platelet counts expressed as *N* × 10^9^/L were compared with the normal range 150 − 450 × 10^9^/L for the determination of thrombocytopenia. Commercially available test strips (Quick Test™ HBsAg Rapid Test Strip, Acon Biotech Diagnostic Systems, San Diego, CA, USA), with relative sensitivity of 99.7% (95% C.I; 99.5%-99.9%) and relative specificity of 99.5% (95% C.I; 97.5%-99.8%), were used to detect the presence HBsAg in the plasma of each participant in accordance with the manufacturer's instructions.

### 2.8. Statistical Analysis

The data was analyzed using SPSS version 22.0 (USA) and Prism 8 (GraphPad, San Diego, USA). Categorical data were represented as frequencies and percentages. Normality check of continuous variables was performed using the Kolmogorov-Smirnov test. Nonparametric data were presented as median (interquartile range) and compared using the Kruskal-Wallis test. Again, bivariate and multivariate logistic regression analyses were utilized to assess associations between categorical variables of interest. *P* value ≤ 0.05 was considered statistically significant.

## 3. Results

### 3.1. Sociodemographic Characteristics and Knowledge of Participants about Hepatitis B

The majority of participants, 128 (94.8%), were <40 years, and the least numbers, 7 (5.2%), were ≥40 years. Also, more than half, 69 (51.1%), were educated to junior high school (JHS) level, whereas the least of them constituting 12 (8.9%) had tertiary education. Majority of the study participants, 100 (74.1%), were employed, with most of them, 121 (89.1%), also being Christians. Again, nearly half of the participants, 66 (48.9%), were single at the time of the study. Assessment of respondents' knowledge about the causes and mode of transmission showed that only 17 (12.6%) had knowledge about the causes, transmission, and prevention of hepatitis B whereas 118 (87.4%) confirmed they had no knowledge about hepatitis B (Table 1).

### 3.2. Gynaecological/Obstetric History of the Respondents

At the time of the study, most of the participants, 63 (46.7%), were in their first trimester, whereas the majority, 69 (51.1%), also confirmed they had history of 2-3 pregnancies. Similarly, the majority representing more than half, 74 (54.8%), had 1-2 children, and the least 21 (15.6%) had 3-5 children until the current study. Again, majority of respondents, 105 (77.8%), had no history of pregnancy termination, whereas a few, 7 (5.2%), had lost ≥2 pregnancies prior to the study (Table 1). Only a few (5%) who had a history of pregnancy termination confirmed that the loss of their babies was associated with prolonged bleeding at delivery.

### 3.3. Sexual Behavior of the Respondents

Over half of the participants, 75 (55.6%), had no history of multiple sexual relationships, whereas 60 (44.4%) confirmed otherwise (Table 2). Among the individuals with history of multiple sexual relationships, the majority (45%) had a maximum of two (2) sexual partners. Also, a minimum, 31 (23%), of the respondents used protection during sexual intercourse whereas the majority, 104 (77%), did not use protection (Table 2). Likewise, about 41% utilized emergency contraceptives, 34% used implants, and 22% utilized condoms, whereas 3% used injectable. This also indicates that most of the respondents were only interested in preventing pregnancy rather than sexually transmitted infections including hepatitis B. In addition, among individuals who used protection, about 74% confirmed they used protection infrequently, whereas 26% used it frequently.

### 3.4. Risk of Exposure of the Respondents

The overwhelming majority representing 129 (95.6%) had no history of intravenous drug use whereas 6 (4.4%) affirmed otherwise. The results again showed that only 2 (1.5%) of the respondents had a tattoo as compared to 133 (98.5%) who had no tattoos (Table 2). Lastly, the results indicated that most of them constituting about 125 (92.6%) were never diagnosed with an STI whereas as a few, 10 (7.4%), had history of an STI.

### 3.5. HBsAg Status, History of Screening, and Vaccination

Prior to the current study, only 14 (10.4%) had history of participating in HBsAg screening whereas 121 (89.6%) had no history of involvement in HBsAg screening. Meanwhile, 9 (6.7%) were vaccinated, while 126 (93.3%) were not vaccinated as at the time of the study. Furthermore, out of the 6.7% of respondents who were vaccinated, 3 (33.3%) completed the vaccination, while 6 (66.67%) did not complete the vaccination as at the time of the study. In the current study, the results from HBsAg screening showed that 129 (95.6%) of the respondents were negative, while 6 (4.4%) of the respondents were positive for HBsAg (Table 2).

### 3.6. Prolonged Bleeding Tendency Assessment

**Figure 1:**
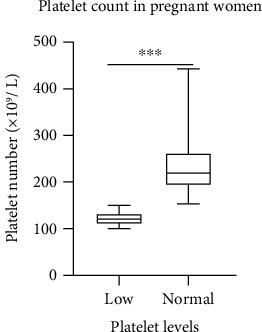
Platelet levels in pregnant women: 19 (14.1%) of the pregnant women had low platelet counts (mild thrombocytopenia) with an average of 121 × 10^9^/L, whereas 116 (85.9%) had normal platelet levels with an average of 236 × 10^9^/L. The data is presented as median (Q1-Q3) and analyzed with the Kruskal-Wallis test; ∗∗∗ represents *P* < 0.00001.

The overwhelming majority, 116 (85.9%), of the respondents had their platelet levels within the normal range, whereas 19 (14.1%) had their platelet levels that were below the normal range (mild thrombocytopenia) (Table 2). There was a statistically significant difference between platelet levels of pregnant women with low count (mild thrombocytopenia) and those with normal platelet counts (*P* < 0.0001) (Figure 1). The bleeding time assay also revealed that 2 (1.5%) of the respondents had a prolonged bleeding time beyond the normal range, whereas 133 (98.5%) had their bleeding time within the normal range (Table 2).

### 3.7. Association of Sociodemographics, History of Screening, and Vaccination with the Knowledge about Hepatitis B

Analysis of the data showed that among pregnant women < 30 years of age, majority (91.6%) had no knowledge about the causes and mode of hepatitis B transmission compared with those ≥ 30 years of age (80.8%), although the age of the respondents was not significantly associated with their knowledge about hepatitis B.

During the bivariate logistic regression analysis, variables like educational status, marital status, screening history, and vaccination history were statistically associated with awareness of hepatitis B. The multivariable logistic regression analysis showed that participants who had tertiary education were about 8 times more likely to be aware of hepatitis B than those without tertiary education (AOR = 7.78, 95% CI: 1.50-40.50). Finally, participants who reported that they previously had hepatitis B screening were about 14 times (AOR = 13.66, 95% CI: 1.72-108.75) more likely to demonstrate knowledge of hepatitis compared to those who have never been screened (Table 3).

## 4. Discussion

In the current study, majority of the pregnant women involved were <40 years of age whereas the least numbers were ≥40 years of age. The sexually active stage in women may be categorized between 18 and 40 years of age [13] that may present with higher risk of acquiring a sexually transmitted infection, including hepatitis B. The predominant age distribution in this study was similar to a recent Ethiopian study which assessed prevalence of hepatitis B in pregnant women assessing antenatal care [14]. The health-associated behavior of an individual could be affected by factors including their knowledge, attitude, and practice [15]. Knowledge, connoting the understanding of individuals about an issue, is crucial in influencing a person's attitude which is the expression of feelings towards that particular issue [15]. In the current study, only 12.6% of pregnant women had knowledge about the causes, mode of transmission, and prevention of hepatitis B as compared with 87.4% who confirmed they had no knowledge about them. Also, the level of education, marital status, history of screening, and vaccination were significantly associated with demonstration of knowledge about hepatitis B. In a recent study among university students, 77.2% demonstrated knowledge about the prevention and transmission of hepatitis B infection, whereas over 75% also demonstrated positive attitude towards measures leading to the prevention of the infection [16]. This confirms the evidence in this current study in which 58.3% of pregnant women with tertiary level of education had knowledge about the causes, transmission, and prevention of hepatitis B as compared with 8.1% of individuals with only pretertiary level of education, who had knowledge about the infection. Participation in screening and vaccination exercises is accompanied with sensitization and education of participants about the infection and how it could be prevented and/or managed. Likewise, most premarital counselling sections have incorporated routine medical examinations or screening for various conditions including STIs which are accompanied with education of couples about various diseases including hepatitis B. Our findings showed that there was low screening (10.4%) and vaccination (6.7%) rates, confirming the evidence in a recent Ugandan study which concluded that low screening and vaccination rates are associated with lack of awareness about hepatitis B [17].

The current study recorded hepatitis B prevalence of 4.4% in the pregnant women. This was close to the prevalence (4.9%) recorded in a recent study among Ethiopian pregnant women [14]. Also, assessment of prolonged bleeding risk showed that 14.1% had thrombocytopenia whereas 1.5% presented with prolonged bleeding time. The current study does not endorse hepatitis B infection as the sole cause of prolonged bleeding risk in pregnant women, since several other confounders could also account for this [18]. However, the evidence only suggests an association of hepatitis B with thrombocytopenia in pregnant women. Thrombocytopenia prevalence was higher compared to a recent study with 9% prevalence among pregnant women [19]. The relatively high prevalence in this study might be due to aggravation by associated risk posed by the hepatitis B viral infection. Quantitative (platelet count) and functional (bleeding time) assays are crucial in assessing disorders of platelets that might predispose individuals to prolonged bleeding risk. Low platelet levels may be attributed to hypersplenism resulting from portal hypertension. Other less common mechanisms of thrombocytopenia in chronic viral hepatitis might be associated with impaired platelet production as a result of suppressed liver synthesis of thrombopoietin, aggravated platelet destruction, and damage to megakaryocytes and platelets by viruses. In this study, the difference between the average number of platelets in patients with thrombocytopenia and normal platelet levels was statistically significant (*P* < 0.0001) and, therefore, may have significant impact on the pregnancy outcome. This requires prompt monitoring and management strategies in ensuring that prolonged bleeding tendencies in pregnancy are averted. However, the findings from the current study may not be representative of all districts and/or regions in the country as the sampling frame was limited to only one center/municipality.

### 4.1. Strengths and Limitations of the Study Findings

The current finding uncovers an important evidence that would not have been routinely assessed in antenatal services at the Suhum Municipality of Ghana. The combination of both questionnaires and laboratory assays was helpful to collect this evidence that could enlighten clinicians in the management and improvement of pregnancy outcomes.

However, as a limitation, the current study was only a one-center study and, therefore, requires validation in other regions as well as among large population groups. Also, the study did not follow up with the assessment of the severity of infection via hepatitis B profiling assay. In addition, the current study was unable to screen for all other confounding clinical conditions associated with thrombocytopenia; therefore, only the notable clinical history of the patients was used as the basis for excluding unqualified participants.

## 5. Conclusion

The prevalence of hepatitis B among pregnant women at the Suhum Municipality of Ghana was 4.4%, with 14.1% prevalence of mild thrombocytopenia and 1.5% prevalence of prolonged bleeding time. Educational level and previous screening history were significantly associated with the demonstration of knowledge about hepatitis B. This suggests that an appreciable proportion of pregnant women with hepatitis B infection might be at risk of poor pregnancy outcome due to thrombocytopenia and prolonged bleeding risk. Further studies should be done in other regional and/or geographic sites, as well as among large population groups, to assess the association of diverse region-specific sociodemographics with knowledge of HBV.

## Figures and Tables

**Table 1:** Sociodemographic, gynaecological, and obstetric history of the respondents.

Variable	Frequency, *n* (%)
Age group (years)	
<20	20 (14.8)
20-24	29 (21.5)
25-29	34 (25.2)
30-34	30 (22.2)
35-39	15 (11.1)
≥40	7 (5.2)
Level of education	
Primary	17 (12.6)
JHS	69 (51.1)
SHS	37 (27.4)
Tertiary	12 (8.9)
Occupation status	
Employed	100 (74.1)
Unemployed	35 (25.9)
Religious affiliation	
Christian	121 (89.6)
Islam	14 (10.4)
Marital status	
Cohabitating	10 (7.4)
Married	59 (43.7)
Single	66 (48.9)
Knowledge of HBV	
Knowledgeable	17 (12.6)
Nonknowledgeable	118 (87.4)
Trimester of pregnancy	
1	63 (46.7)
2	57 (42.2)
3	15 (11.1)
No. of pregnancies	
1	34 (25.2)
2-3	69 (51.1)
4-5	23 (17.0)
6-9	9 (6.7)
Child birth history	
0	40 (29.6)
1-2	74 (54.8)
3-5	21 (15.6)
Pregnancies lost	
0	105 (77.8)
1	23 (17.0)
≥2	7 (5.2)

The data is presented as frequencies, *n* (%).

**Table 2:** Hepatitis B and prolonged bleeding tendency assessment.

Assessment of risk factors and screening assays	Frequency, *n* (%)	
	Yes	No
Sexual behavior		
Multiple sexual relations	60 (44.4)	75 (55.6)
Utilized protection	31 (23.0)	104 (77.0)
Risk of exposure		
Intravenous drug use	6 (4.4)	129 (95.6)
Presence of tattoos	2 (1.5)	133 (98.5)
History of STI	10 (7.4)	125 (92.6)
HBsAg status and vaccination		
HBsAg screening history	14 (10.4)	121 (89.6)
Vaccinated for HBsAg	9 (6.7)	126 (93.3)
HBsAg positive status	6 (4.4)	129 (95.6)
Prolonged bleeding tendency assessment		
Low platelet count (thrombocytopenia)	19 (14.1)	116 (85.9)
Prolonged bleeding time	2 (1.5)	133 (98.5)

The data is presented as frequencies, *n* (%).

**Table 3:** Bivariate and multivariate logistic regression analyses of factors associated with awareness of hepatitis B.

Categories	Knowledge of hepatitis B, *n* (%)		COR (95% CI)	*P* value	AOR (95% CI)	*P* value
	Yes	No				
Age group						
<30 years	7 (8.4)	76 (91.6)	1		1	
≥30 years	10 (19.2)	42 (80.8)	2.59 (0.92-7.29)	0.073	0.83 (0.21-3.36)	0.795
Educational level						
Pretertiary	10 (8.1)	113 (91.9)	1		1	
Tertiary	7 (58.3)	5 (41.7)	15.82 (4.24-59.06)	<0.001	7.78 (1.50-40.50)	0.015
Marital status						
Single/cohabitation	5 (6.6)	71 (93.4)	1		1	
Married	12 (20.3)	47 (79.7)	3.63 (1.20-10.96)	0.023	1.79 (0.40-8.06)	0.446
Screening history						
Never screened	9 (7.4)	112 (92.6)	1		1	
Previously screened	8 (57.1)	6 (42.9)	16.59 (4.72-58.36)	<0.001	13.66 (1.72-108.75)	0.014
Vaccination history						
Not vaccinated	11 (8.7)	115 (91.3)	1		1	
Vaccinated	6 (66.7)	3 (33.3)	20.91 (4.58-95.38)	<0.001	0.82 (0.06-10.46)	0.877

The categorical data is presented as frequencies, *n* (%), and analyzed using bivariate and multivariate logistic regression tests. COR: crude odds ratio; AOR: adjusted odds ratio; 95% CI: 95% confidence interval.

## Data Availability

The data supporting the conclusions of the study would be made available to readers upon request from the corresponding author.
